# Multifrequency nonlinear model of magnetic material with artificial intelligence optimization

**DOI:** 10.1038/s41598-022-23810-9

**Published:** 2022-11-17

**Authors:** J. Pawłowski, K. Kutorasiński, M. Szewczyk

**Affiliations:** 1 Department of Theoretical Physics, Faculty of Fundamental Problems of Technology, Wroclaw University of Science and Technology, Wyb. Wyspiańskiego 27 St., 50-370 Wrocław, Poland; 2 Department of Condensed Matter Physics, Faculty of Physics and Applied Computer Science, AGH University of Science and Technology, Reymonta 19 St., 30-059 Kraków, Poland; 3 Division of Power Apparatus, Protection and Control, Faculty of Electrical Engineering, Electrical Power Engineering Institute, Warsaw University of Technology, Koszykowa 75 St., 00-662 Warszawa, Poland

**Keywords:** Electrical and electronic engineering, Electronic devices

## Abstract

Magnetic rings are extensively used in power products where they often operate in high frequency and high current conditions, such as for mitigation of excessive voltages in high-power switchgear equipment. We provide a general model of a magnetic ring that reproduces both frequency and current dependencies with the use of artificial intelligence (AI) optimization methods. The model has a form of a lumped element equivalent circuit that is suitable for power system transient studies. A previously published conventional (non-AI) model, which we take as a starting point, gives a good fit of parameters but uneven characteristics as a function of current, which pose numerical instabilities in transient simulations. We first enforce the Langevin function relationship to obtain smooth characteristics of parameters, which reduces the number of parameters and ensures their even characteristics, however, compromises fit quality. We then use AI metaheuristic optimization methods that give a perfect fit for the model in the whole range of frequency up to 100 MHz and current up to saturation, with smooth characteristics of its parameters. Additionally, for such fitted parameters, we show that it is feasible to find a frequency dependence for the magnetic saturation parameter of the Jiles-Atherton (JA) model, thus enabling frequency-dependent JA.

## Introduction

Magnetic materials are extensively used in electrical power products, not only for Electromagnetic Interference (EMI) suppression, but also for^1^ mitigation of overvoltages in electronics and electrical power devices, such as power electronics converters, motors and generators, and Gas Insulated Switchgear (GIS). Oftentimes, the magnetic rings operate in a single-turn arrangement, e.g., the rings installed onto the shaft of motors to mitigate bearing currents^2^, or the rings installed onto the GIS busbars for mitigation of Very Fast Transient Overvoltages (VFTO)^3,4^, or for High Frequency Transients mitigation in wind turbine transformers^5^. Selection of the magnetic rings with a proper material characteristics and geometry, matching the type of the material and its quantity to the problem, requires mathematical models usable in practical simulation frameworks.

## Magnetic rings modeling

### Frequency and current characteristics

For linear conditions (i.e., when the system response is a linear function of the forcing magnetic field $H$), full information on magnetic system is enclosed in the impedance $Z(f)$ in frequency $f$-domain, or the equivalent transfer function $Z(s)$ in s-domain, which in the case of a coil is a transfer function of current to voltage. The $Z(f)$ results from underlying, often very complex, physics of a given material^6^. Coupling between spins in the Ising model or Curie temperature (determining material type)^6^ or domain walls movements (determining frequency response and magnetic losses)^7^, are complex many-body phenomena with long story of research and various physical models^8,9^. Considering the $Z(f)$ characteristics we enclose in this curve all the physics necessary for power system transients simulations (black box approach). From $Z(f)$ other parameters can be calculated, such as inductance $L(f)$ and resistance $R(f)$ of, e.g., a series $RL$ model, permeability $\mu (f)$, or power losses $P(f)$.

In the case of nonlinear systems, the situation is more complex. First, the harmonic response for a nonlinear system is not defined. A natural approximation of nonlinear systems is the assumption of their linear operation in a small range of the forcing signal (i.e., small enough for the system to respond linearly). This allows to define a complex impedance $Z(f, {H}_{\mathrm{bias}})$, which accurately describes the behavior of nonlinear magnetic systems up to saturation, in which the forcing magnetic field $H(i)$ is of a form:

1 $$ H\left( i \right) = H\left( {i_{{{\text{AC}}}} } \right) + H_{{{\text{bias}}}} \left( {i_{{{\text{DC}}}} } \right) $$

where $H\left( {i_{{{\text{AC}}}} } \right)$ is the small forcing magnetic field induced by a small $i_{{{\text{AC}}}}$ current (i.e., small enough for the system to respond linearly), and $H_{{{\text{bias}}}} \left( {i_{{{\text{DC}}}} } \right)$ is the magnetic field induced by a direct current $i_{{{\text{DC}}}}$ which establishes the operating condition of magnetic ring up to saturation. For magnetic rings operating in one-wire set-up:

2 $$ H_{{{\text{bias}}}} \left( {i_{{{\text{DC}}}} } \right) = \frac{{l_{{\text{m}}} }}{{A_{{\text{e}}} }}i_{{{\text{DC}}}} $$

where ${l}_{m}$ is magnetic path in meters, and ${A}_{\mathrm{e}}$ is the cross-sectional area of the ring in square meters. In this paper we use a notation where $Z(f,{H}_{\mathrm{bias}}({i}_{\mathrm{DC}}))$ is written as $Z(f,{i}_{\mathrm{DC}})$. The function $Z(f,{i}_{\mathrm{DC}})$ is the response of the magnetic system at the value of ${H}_{\mathrm{bias}}({i}_{\mathrm{DC}})$, setting the so-called operating point. The nonlinear system is then approximated by a two-dimensional function $Z(f,{i}_{\mathrm{DC}})$.

The function $Z(f,{H}_{\mathrm{bias}})$ can be measured using an impedance analyzer that generates the magnetic field $H({i}_{\mathrm{AC}})$ supplemented with a $DC$ power supply providing ${i}_{\mathrm{DC}}$ current to set the ${H}_{\mathrm{bias}}({i}_{\mathrm{DC}})$ operating point. This measurement method was introduced in^10^ and the results of the measured $Z (f,{i}_{\mathrm{DC}})$ impedance reported in^10^ were used in the present study.

### Magnetic materials modelling

Various models are in use to modeling of magnetic materials in nonlinear conditions. One most notable is the Jiles-Atherton (JA) model^11,12^ of nonlinear ordinary differential equations for magnetization, defined to describe magnetic hysteresis. Second family of models, Finite Element Method (FEM)-based describe local spatial dependence of magnetization^13^. The JA model can be coupled with the FEM^14,15^, extending the latter approach onto nonlinear materials. On the other hand, a different approach to modeling of magnetic materials uses the lumped element equivalent circuits (LEEC). The LEEC approach is suitable for direct implementation in SPICE or EMTP simulators^16^ which enables fast prototyping.

In this paper we apply Artificial Intelligence (AI) algorithms for optimizing of a LEEC model parameters of magnetic ring. For this we use metaheuristic optimization methods with Particle Swarm Optimization (PSO) and Differential Evolution (DE) and modern implementation of simulated Dual Annealing (DA).

## Nonlinear Lumped Elements model

LEEC are typically used to modeling linear responses of magnetic rings in frequency domain, but when lumped elements are extended to nonlinear characteristics the LEEC can be efficiently used to modelling of magnetic rings in nonlinear conditions as well^10,17^. The great advantage of such approach is that it is capable to modeling of magnetic rings in both frequency and current domains, and in a form that can be directly implemented in circuit simulators such as EMTP or SPICE^16^. The approach was used in^10^, where the LEEC model was proposed that reproduced the impedance characteristics $Z(f,i)$ of magnetic ring in both frequency and current domains.

In this paper, we propose frequency- and current-dependent models of magnetic rings in a form of $Z(f, i)$, where $i=i(t)$ is any current, but the models are based on measured $Z(f, {H}_{\mathrm{bias}})$. Such models are strictly accurate for currents $i(t) = {i}_{\mathrm{AC}} + {i}_{\mathrm{DC}}$. Generalization of the models for any current $i=i(t)$ is an extrapolation of the model beyond the data on which it was created. The scope of applicability of this extrapolation is not investigated in this paper.

A dedicated test set-up was proposed in^10^ allowing to measure $Z(f)$ for different bias magnetic field ${H}_{\mathrm{bias}}({i}_{\mathrm{DC}})$, introduced to the magnetic ring by the bias direct current ${i}_{\mathrm{DC}}$ of a reference coil. The impedance characteristics $Z(f,{i}_{\mathrm{DC}})$ was thus formed by a set of $Z(f)$ functions measured for different values of the bias current ${i}_{\mathrm{DC}}$ defining different operating conditions.

**Figure 1:**
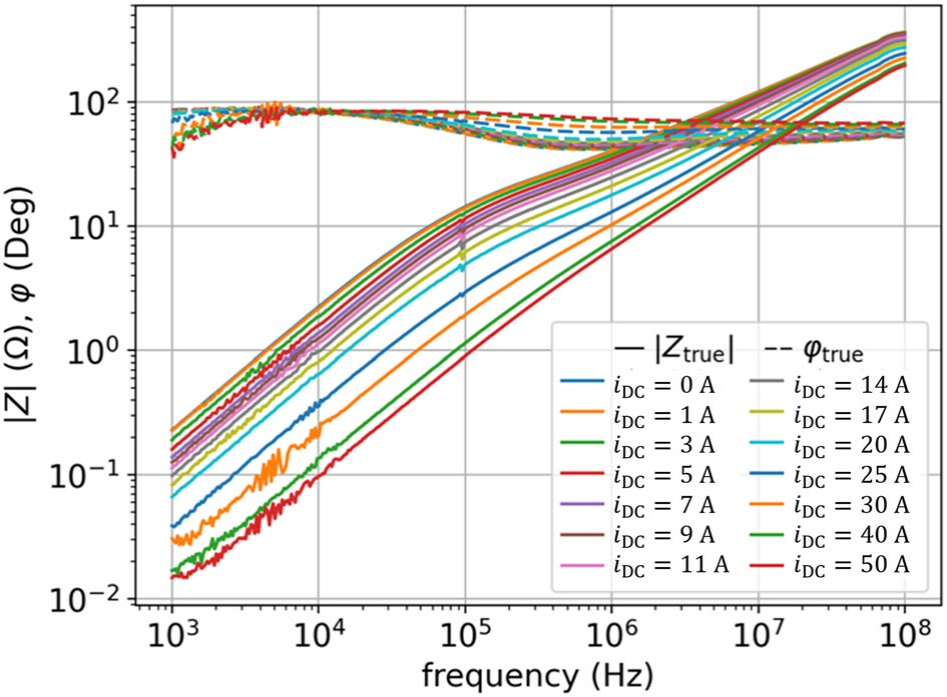
Experimental data from^10^ used in this paper for fitting parameters of models: measured $Z_{{{\text{true}}}} \left( {f,i_{{{\text{DC}}}} } \right)$ impedance characteristics for frequency range $f = 1 {\text{kHz}} \div 100 {\text{MHz}}$ and current up to saturation $i = 0 \div 50 {\text{A}}$; solid lines denote absolute values $\left| {Z_{{{\text{true}}}} } \right|\left( {f,i_{{{\text{DC}}}} } \right)$ and dashed lines denote phase angles $\varphi_{{{\text{true}}}} \left( {f,i_{{{\text{DC}}}} } \right)$; colors denote bias current $i_{{{\text{DC}}}}$ according to legend.

Figure 1 shows the $Z(f,{i}_{\mathrm{DC}})$ characteristics measured in^10^ for a nanocrystalline magnetic ring with the $DC$ current ${i}_{\mathrm{DC}}$ ranging from zero up to saturation level. We use in this paper the $Z\left(f,{i}_{\mathrm{DC}}\right)$ curves from^10^ as shown in Fig. 1 to investigate and propose new LEEC models of magnetic ring extending the model reported in^10^. The noise visible for frequencies below ${10}^{4} \mathrm{Hz}$ results from the measurement of impedance $|Z|$ (see Fig. 1), which decreases as frequency $f$ decreases, which causes that for small $f$ there is a large ratio of noise in $|Z|$ to the measured value.

### Lumped model

**Figure 2:**
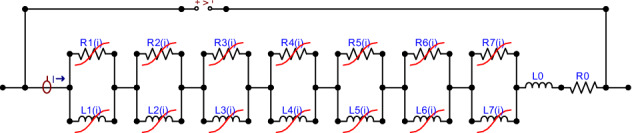
Lumped Element Equivalent Circuit (LEEC) model given by formula (3) proposed in^10^ to reproduce $Z\left(f,{i}_{\mathrm{DC}}\right)$ characteristics shown in Fig. 1.

The idea of the LEEC model of magnetic coil introduced in^10,17^ is to approximate a frequency response function $Z(f)$ with a rational function $Z(s)$ in $s$-domain in a form having equivalent electrical circuit representation. The approximation is performed jointly for several operating conditions given by $Z(f)$ for different currents ${i}_{\mathrm{DC}}$. The network is synthetized in a standard Foster form^18^. This way the system impedance $Z\left(f,{i}_{\mathrm{DC}}\right)$ approximates $Z\left(f,i\right)$, $Z\left(f,{i}_{\mathrm{DC}}\right)=Z\left(f,i\right)$, and is modeled by a rational function:

3 $$ Z\left( {s,i} \right) = \mathop \sum \limits_{k = 1}^{{N_{l} }} \frac{{sR_{k} \left( i \right)}}{{s + R_{k} \left( i \right)/L_{k} \left( i \right) }} + sL_{0} + R_{0} , $$

with nonlinear ${L}_{k}(i)$ and ${R}_{k}(i)$ elements representing different operating conditions given by current ${i}_{\mathrm{DC}}$. The ${L}_{0}$ and ${R}_{0}$ elements allow for a general $LR$ model of impedance, such as the one proposed in^19^. For the transfer function of a dynamical system, where the numerator degree must be less than or equal to the degree of the denominator in the general rational function, the two components, ${L}_{0}$ and ${R}_{0}$, can be skipped. In^10^ the network was synthetized with ${N}_{l}=7$ pairs of ${L}_{k}(i)$ and ${R}_{k}(i)$ lumped elements, each of which having nonlinear characteristics for ${N}_{i}=14$ operating conditions given by current ${i}_{\mathrm{DC}}$ values as denoted in Fig. 1, ranging from $0 \mathrm{A}$ up to saturation. In the present work, we chose the same number ${N}_{l}=7$ as in^10^, which was here confirmed based on trial and error as a kind of optimization, in which, on the one hand, we wanted the model to be as simple as possible and contain as few elements as possible, and on the other hand, that the fit should be of good quality – in the entire frequency domain and for all currents. The increase in the number of parameters did not significantly improve the fit, and the decrease made it worse. The resultant network is shown in Fig. 2.

In formula (3) all poles ${p}_{k}(i)=-{R}_{k}(i)/{L}_{k}(i)$ are stable, i.e., ${p}_{k}(i)<0$ for all $i={i}_{\mathrm{DC}}$ and all $k$. To fit parameters, a method based on Pade approximation was used in^10^ as described in^17^.

Fit on the ${L}_{k}(i)$ and ${R}_{k}(i)$ ladders connected in parallel allows to model systems which phase is in the range of $0$-$90$ deg only, i.e., inductive-resistive systems. Additionally, if we assume that we will use only positive values of the elements ${L}_{k}(i)$, ${L}_{0}$ and ${R}_{k}(i)$, ${R}_{0}$ – then it leads to an additional condition that the circuit must have a non-decreasing impedance as a function of frequency. These conditions are valid for the characteristics of magnetic rings where capacitive components are negligible. In the measured impedance frequency characteristics $Z(f,{H}_{\mathrm{bias}})$ no resonances are observed (see Fig. 1).

When the LEEC model (3) reproduces the $Z(f,i)$ characteristics in a wide frequency range (implying high ${N}_{l}$) and for many currents ${i=i}_{\mathrm{DC}}$ (implying high ${N}_{i}$), the number of necessary parameters may reach hundreds: $2{N}_{l}{N}_{i}$+2. In the case of $Z(f,{i}_{\mathrm{DC}})$ given by Fig. 1 for ${N}_{i}=14$ operating currents: $2{N}_{l}{N}_{i}+2= 2\cdot 7\cdot 14+2=198$ parameters in total. The number of parameters is large because we have a large number of operating currents ${i}_{\mathrm{DC}}$, and we adjust the $Z(f)$ for each operating current ${i}_{\mathrm{DC}}$ independently. In general, the ${N}_{i}$ can be even much higher when higher resolution of ${i}_{\mathrm{DC}}$ would be applied, so the current $i={i}_{\mathrm{DC}}$ can be considered as a second dimension of the model. The number of parameters can be further reduced (by half) assuming linear resistance of each ${R}_{k}$ element, i.e., ${R}_{k}\left(i\right)=\mathrm{const}$. The magnetic loses depend on the operating current $i$ and decrease when entering saturation, as they result from remagnetization of magnetic domains (while the domains are more firmly fixed by the external bias magnetic field $H$, the losses decrease). However, as seen in Fig. 4, the ${R}_{k}(i)$ is significantly less current-dependent then ${L}_{k}(i)$. In our case, assuming ${R}_{k}\left(i\right)=\mathrm{const}$ does not deteriorate the optimization results (fit error), and significantly simplifies the model.

Another limitation of this model is that it does not guarantee that the parameters ${L}_{k}(i)$ and ${R}_{k}(i)$ will be smooth or even continuous functions of current $i$, which leads to unphysical behavior related to numerical problems during simulations. We will aim at avoiding a situation shown in Fig. 4 with this respect.

### Parameters fitting

Vector Fitting (VF)^19,20^ algorithm is a popular procedure for s-domain rational function approximation of frequency-domain characteristics. In VF, the poles are enforced to be stable (i.e., with nonpositive real part), but the algorithm cannot guarantee that the identified function is a positive real function (i.e., it represents a passive system)^21^. Passivity is important to get stable simulations in time domain, e.g., in transient simulations. In our case, the VF cannot ensure that ${L}_{k}(i)$ and ${R}_{k}(i)$ are *both* non-negative. Moreover, in a vector form of ${L}_{k}(i)$ and ${R}_{k}(i)$, i.e., for simultaneous fit of many $Z(f)$ characteristics (in our case $Z(f,i)$ formed by a set of $Z(f)$ for different currents $i$), the VF forces the same poles ${p}_{k}(i)=-{R}_{k}(i)/{L}_{k}(i)$, which is too much of a limitation to get a good fit. Another disadvantage of this approach is that it does not guarantee the passivity of the fitted lumped elements ${R}_{k}(i)$, ${L}_{k}(i)$, ${R}_{0}$, ${L}_{0}$.

The VF is not suitable for fitting ${L}_{k}(i)$ and ${R}_{k}(i)$ ladders also because it does not guarantee that the order of the s-domain rational function $Z\left(s,i\right)$ will not be higher than $1$ (e.g., 2nd order corresponds to a system with capacitance). The VF is a general approach not assuming that the phase of a particular element of the ladder (${L}_{k}(i)$ and ${R}_{k}(i)$ for a given $k$) is in the range of $0$-$90$ deg, only that the fit will be good. Thus, it does not guarantee that the circuit representation of the resulting s-domain function $Z(s,i)$ has a ladder representation as we demand.

In^10^, the Pade approximation method was used with a properly chosen auxiliary function proposed in^17^, allowing to properly represent experimental data $Z(f,{i}_{\mathrm{DC}})$. In both the VF and the Pade based methods, however, the fits of $Z(f)$ are made for individual operating currents ${i}_{\mathrm{DC}}$ independently, which gives no guarantee that ${L}_{k}(i)$ and ${R}_{k}(i)$ will change smoothly as a function of current $i$. This is not suitable for simulations, for which both discontinuous and non-decreasing ${L}_{k}(i)$ and ${R}_{k}\left(i\right)$ parameters in the s-domain rational function $Z(s,i)$ pose a problem.

### Nonlinear least squares optimization

Therefore, we start fitting and optimizing the parameters ${L}_{k}\left(i\right)$, ${R}_{k}(i)$, ${L}_{0}$ and ${R}_{0}$ of (3) from a standard Nonlinear Least Squares (NLS) method (nonlinear, as there are parameters also in the denominator), on which appropriate bounds can be imposed^22^ – in our case non-negativity of $L$ and $R$ elements. In the NLS we choose Trust Region Reflective (TRR) method to perform minimization and the so-called robust soft-L_1_ regularization^22^. We will show disadvantages of this method and then we will gradually modify the model and the method of parameters fit to develop a good fit and smooth characteristics of $L$ (${L}_{k}$ and ${L}_{0}$) and $R$ (${R}_{k}$ and ${R}_{0}$) elements, with smallest number of parameters.

**Figure 3:**
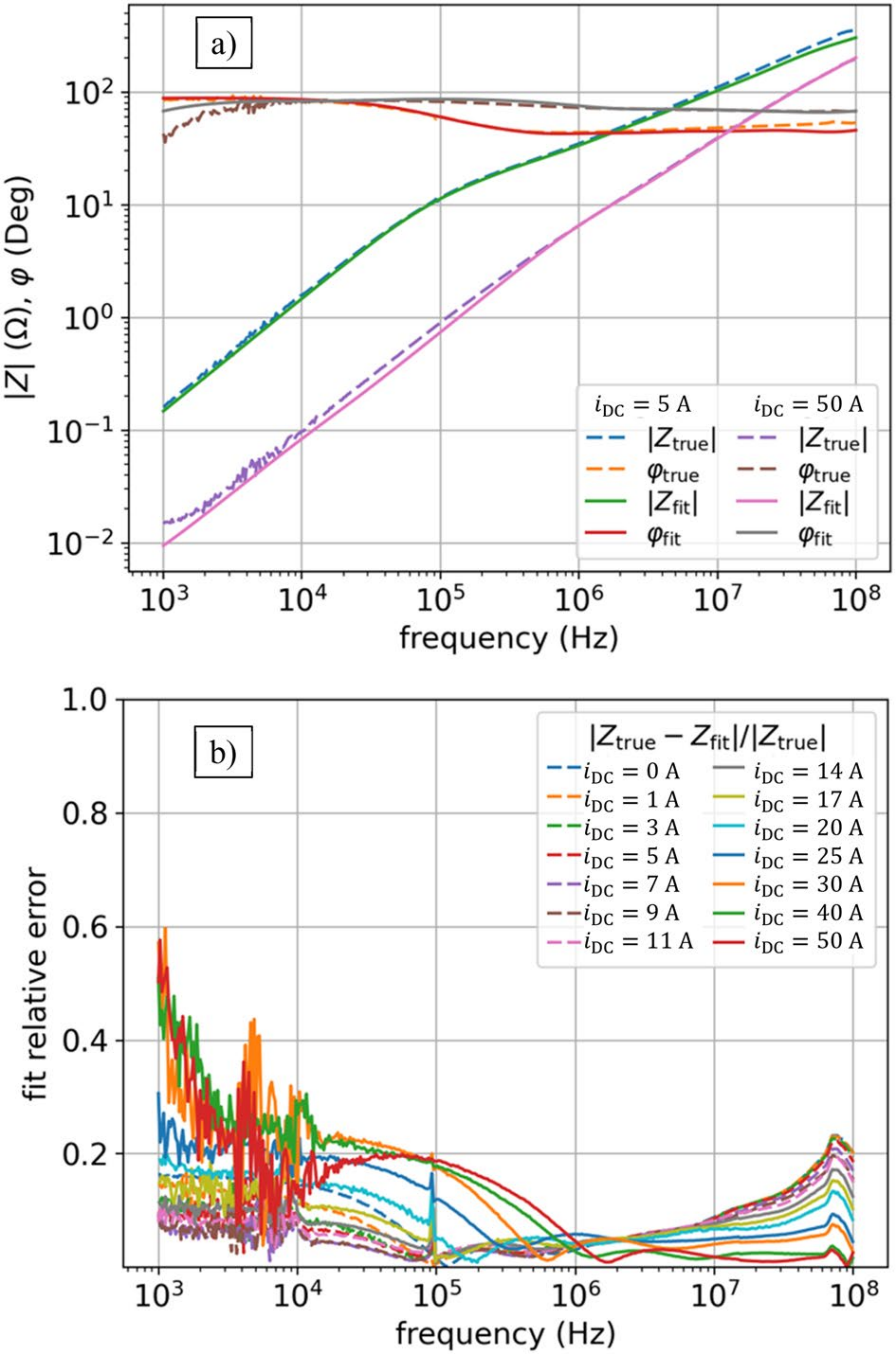
NLS results of impedance $Z(f,{i}_{\mathrm{DC}})$ fitting for model (3): (**a**) fit vs true values of $\left|Z\right|\left(f,{i}_{\mathrm{DC}}\right)$ and $\varphi (f,{i}_{\mathrm{DC}})$ for two currents: ${i}_{\mathrm{DC}}=5$ A, ${i}_{\mathrm{DC}}=50$ A; (**b**) relative error as per (4) for all currents.

To get a good fit we also assume that ${L}_{0}$ and ${R}_{0}$ in (3) are nonlinear, so now in (3) the $s{L}_{0}+{R}_{0}$ turns into ${s{L}_{0}\left(i\right)+R}_{0}(i)$, hence the function (3) now have $\left(2\times {N}_{l}+2\right)\times {N}_{i}=\left(2\times 7+2\right)\times 14 = 224$ parameters. Figure 3a shows the results of fitting, which is good, however not ideal (see also Table 1). The error of the impedance model ${Z}_{\mathrm{fit}}\left(s,i\right)$ is shown in Fig. 3b, calculated for each operating point (each current ${i}_{\mathrm{DC}}$) as defined by:

4 $$ \frac{{|Z_{{{\text{true}}}} \left( {s,i} \right) - Z_{{{\text{fit}}}} \left( {s,i} \right)|}}{{|Z_{{{\text{true}}}} \left( {s,i} \right)|}}, $$

where the measured data (shown in Fig. 1) are represented with ${Z}_{\mathrm{true}}\left(s,i\right)={\left|Z\right|}_{i}{(f)e}^{j{\phi }_{i}(f)},$ where $s=j2\pi f$, for different frequencies $f$, and different operating points given by currents ${i}_{\mathrm{DC}}$. We observe the highest error in the range of the smallest frequencies, then it decreases, and for the largest frequencies it increases again.

**Table 1:** fit relative error^1^ averaged over all frequencies and currents for different optimization methods.

Method	Average error
Nonlinear Least Squares (NLS)	0.286
Particle Swarm Optimization (PSO)	0.196^2^
Differential Evolution (DE)	**0.084**
Dual Annealing (DA)	0.149

^1^see formula (4), ^2^the PSO error is unacceptable.

for higher values of currents *i* ≥ 30 A (see Fig. 7).

Best result is in bold.

**Figure 4:**
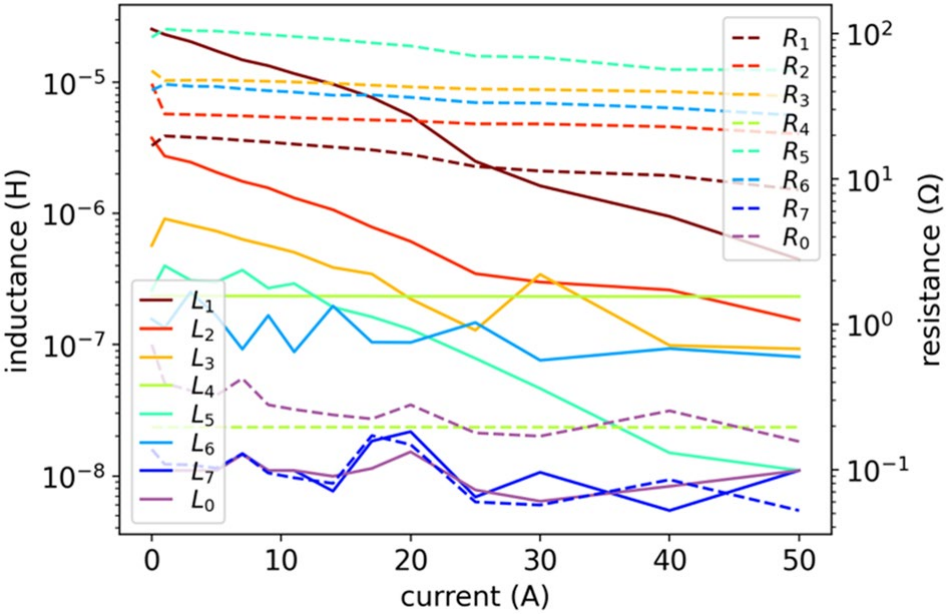
NLS results of inductances $L(i)$ and resistances $R(i)$ in model (3). Here we do not enforce monotonic inductance using Langevin derivative and constant resistance.

The key problem in this approach lies in dependencies of $L(i)$ and $R(i)$ parameters on current $i$, which is shown in Fig. 4. The characteristics $L(i)$ and $R(i)$ are approximately monotonic, but uneven. The NLS numerical method matches $L(i)$ and $R(i)$ independently for each current ${i}_{\mathrm{DC}}$, so numerically there is not any correlation between the fits for different currents, which results in the uneven $L(i)$ and $R(i)$ characteristics as a function of current $i$. This may also lead to numerical problems for such a circuit with uneven parameters when using the LEEC model in EMTP or SPICE simulators.

## Langevin function for nonlinearity modelling

We observe that NSL with the constraints as assumed above fits parameters for LEEC model (3) with current dependent $L(i)$, ${R}_{k}(i)$, ${L}_{0}(i)$, and ${R}_{0}(i)$ well (see Fig. 3), covering wide range of $(f,i)$-domains. However, this needs over 200 parameters and leads to non-smooth characteristics of $L(i)$ and $R$(i) in $i$-domain.

Now we apply constraints on current dependence of inductance $L(i)$ using derivative of Langevin function: $\mathcal{L}\left(x\right)=\mathrm{coth}\left(x\right)-1/x,$ that is used when discussing paramagnetism^8^, and it is also assumed in JA model^11,12^. We introduce analytical characteristics for current dependencies for two reasons: 1) they have a clear physical interpretation, 2) we substantially reduce the number of parameters.

### Langevin model for nonlinearity

We assume a simplest physical model for anhysteretic magnetization^6^ and conversely assume derivative of Langevin (DL) function $\mathcal{L}\mathcal{^{\prime}}\left(x\right)$ ^23,24^ for nonlinear characteristics of inductance:

5 $$ L^{DL} \left( i \right) = l_{0} {\mathcal{L}^{\prime}}\left( {i/a } \right), $$

with only two parameters needed: ${l}_{0}$ – a maximum inductance, and $a$ – scaling factor. The DL derivative we implemented as follows ^23^: $L\left( x \right) = 1/3 - x^{2} /15 + 2x^{4} /189{ }{-}x^{6} /675 + 2x^{8} /11686$ for $x{ } < { }1$, and $L\left( x \right) = { }1/x^{2} - 1/\sinh^{2} (x)$ elsewhere.

Magnetic losses refer to various energy dissipation mechanisms taking place when a magnetic material is subject to a time-varying external magnetic field $H$. To simplify the model and not compromising optimization quality, we assume that energy losses, and thus resistances ${R}_{k}$ and ${R}_{0}$ no longer depend on operating point (on current $i={i}_{DC}$). We verified that this assumption does not lead to a significant increase in the fitting error and may be related to the fact that magnetic losses depend much less on the current than the coil inductance itself (see Fig. 4). On the other hand, losses strictly depend on frequency, which we consider. Now the model is defined by:

6 $$ Z\left( {s,i} \right) = \mathop \sum \limits_{k = 1}^{{N_{l} }} \frac{{sR_{k} }}{{s + R_{k} /L_{k}^{DL} \left( i \right) }} + sL_{0}^{DL} \left( i \right) + R_{0} . $$

Thanks to this, we significantly reduce the number of parameters, which is now $\left(7 + 1\right)\cdot 3=24$: each $L$ has two parameters: ${l}_{0}$ and $a$, and each $R$ represents one parameter (as it is constant). Additionally, it is very important that the number of parameters does not depend on the resolution of the $Z(f)$ measurements as a function of current $i$ (as it was before).

### Nonlinear least squares optimization

**Figure 5:**
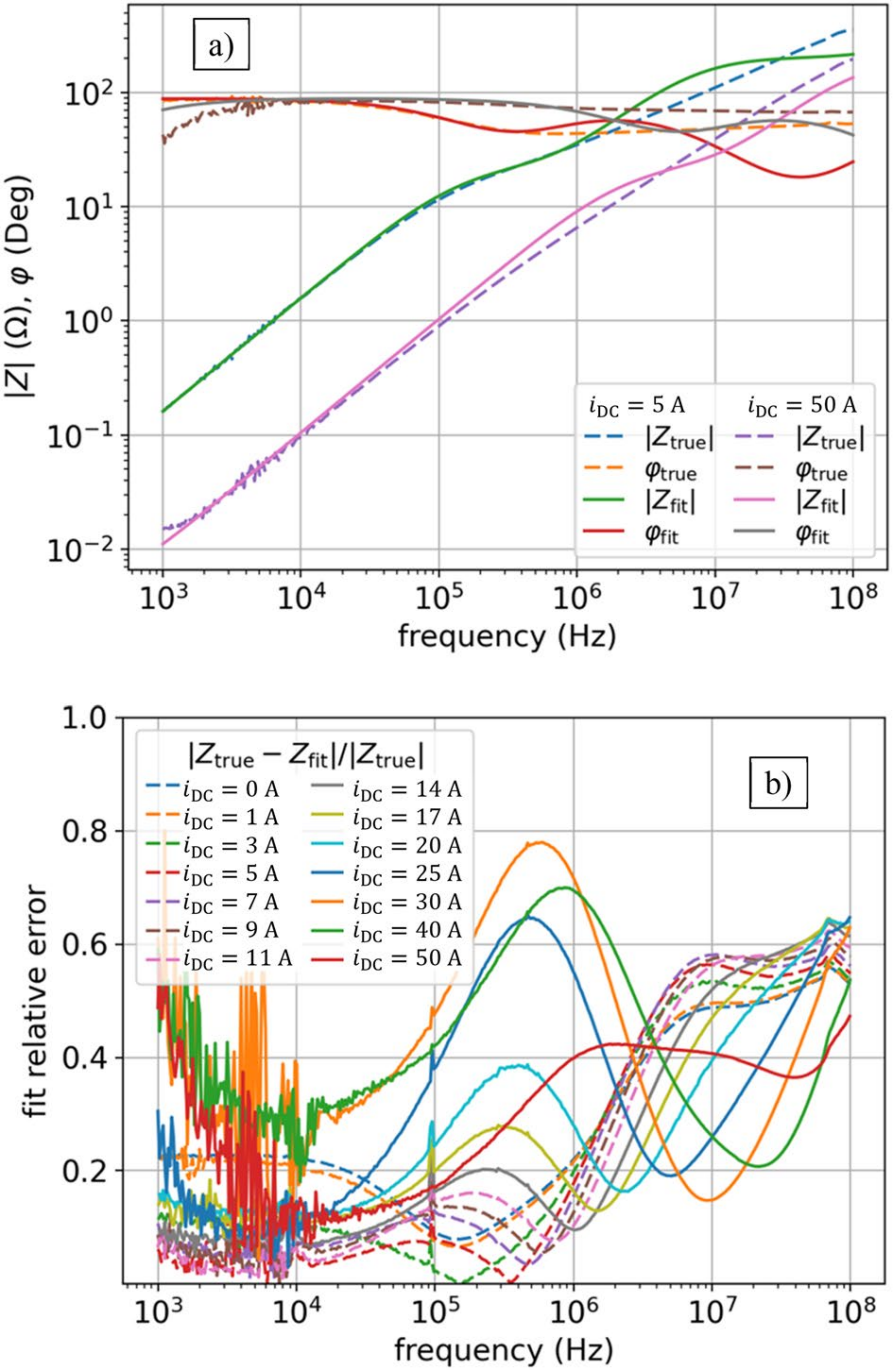
NLS with Langevin constraint results of impedance $Z(f,{i}_{\mathrm{DC}})$ fitting for model (6): (**a**) fit versus true values of $\left|Z\right|\left(f,{i}_{\mathrm{DC}}\right)$ and $\varphi (f,{i}_{\mathrm{DC}})$ for two currents: ${i}_{\mathrm{DC}}=5$ A, ${i}_{\mathrm{DC}}=50$ A; (**b**) relative error as per (4) for all currents. Enforcing monotonic inductances and constant resistances make optimization extremally hard. Best result after many trials for NLS with Langevin constraint gives unacceptable fitting results.

**Figure 6:**
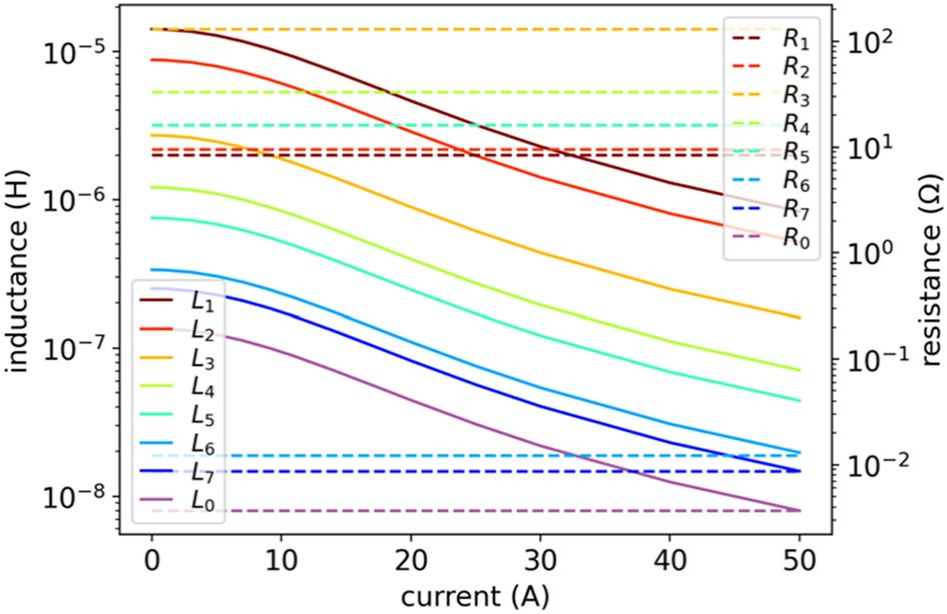
NLS with Langevin constraint results of inductances $L(i)$ given by (5) and constant resistances $R(i)$ in model (6). Langevin-derivative-like inductances and constant resistances are applied.

Now we optimize the new model (6) using NLS and we get nice smooth $L(i)$ characteristics as shown in Fig. 6. On the other hand, though, giving the constraints in the form of D-Langevin (5) makes the fit deteriorated as shown in Fig. 5. The NLS does not cope in this regard: we either made the objective function too complicated or the current dependence of $L(i)$ cannot be properly described by ${L}^{DL}(i)$ function. It is clear that we need some other methods to optimize the parameters for this model, beyond simple NLS.

## Artificial intelligence optimization

Model (6) with inductances ${L}_{k}(i)$ and ${L}_{0}(i)$ in the form (5) has smooth $L(i)$ and $R\left(i\right)$ characteristics (see Fig. 6) and reduced number of parameters in relation to the original model (3) ($24$ in our case as compared to $198$). Moreover, the number of parameters in the original model (3) depends on the resolution of current ${i}_{\mathrm{DC}}$, and in the new model it relates only to the number of ladder elements. However, adding a constraint in the form (5) makes the optimization of this model (parameter selection) much more difficult and results in them giving smooth $L(i)$ and $R(i)$ parameters (as shown in Fig. 6), but having a poor fit (as shown in Fig. 5). We have a good model (6) with good physical interpretation, but we cannot fit its parameters. We need better optimization techniques to restore the model usability. Thus, we need better parameters optimization technique to restore the model usability.

We will now use and compare two modern approaches to multiparameter optimization: evolutionary algorithms (see Section V.A) and simulated annealing method (see Section V.B).

The evolutionary algorithms are part of artificial intelligence-based computational techniques designed mainly for global optimization inspired by biological evolution. We will use two evolutionary approaches: Particle Swarm Optimization (PSO)^25,26^ and Differential Evolution (DE)^27,28^.

The simulated annealing^29,30^ is an optimization method with inspiration coming from annealing in metallurgy. At each time step, the algorithm randomly selects a solution close to the current one, measures its quality, and moves to it according to the temperature-dependent probabilities of selecting better or worse solutions. Accepting worse solutions allows for a more extensive search for the global optimal solution. We will use modern implementation of simulated annealing: Dual Annealing (DA) algorithm^31^.

All three methods (PSO, DE, and DA) are metaheuristics. Metaheuristics typically make few or no assumptions about the problem being optimized and can search very large (much larger than traditional methods) spaces of candidate solutions. However, metaheuristics do not guarantee an optimal solution is ever found. But we will show that in our case these methods will be much better than traditional NLS.

### Evolutionary optimization

**Figure 7:**
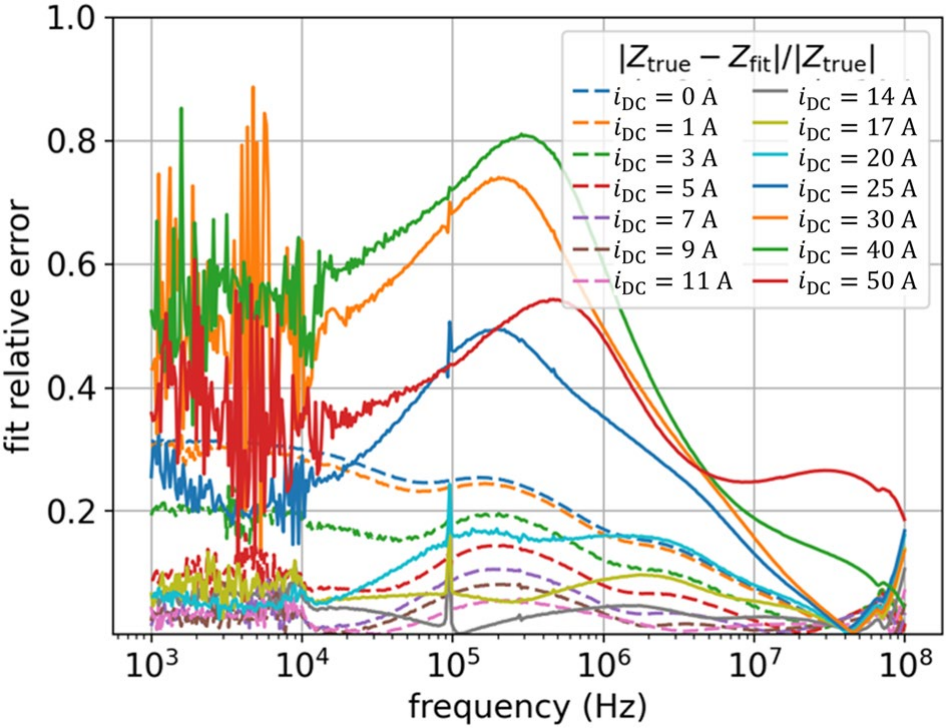
PSO results of impedance $Z(f,{i}_{\mathrm{DC}})$ fitting for model (6): relative error as per (4) for all currents. Unacceptable fitting results.

In PSO a population of candidate solutions (particles) are moved around in the search-space according to a mathematical formula for the particle's position and velocity. Each particle's movement is influenced by its local best-known position but is also guided towards the best-known positions in the search-space. Figure 7 shows the results for the PSO: a characteristic problem was that it was not feasible to find a fit in a wide range of currents, e.g., for $i\ge 30 \mathrm{A}$ the fit is already weak.

**Figure 8:**
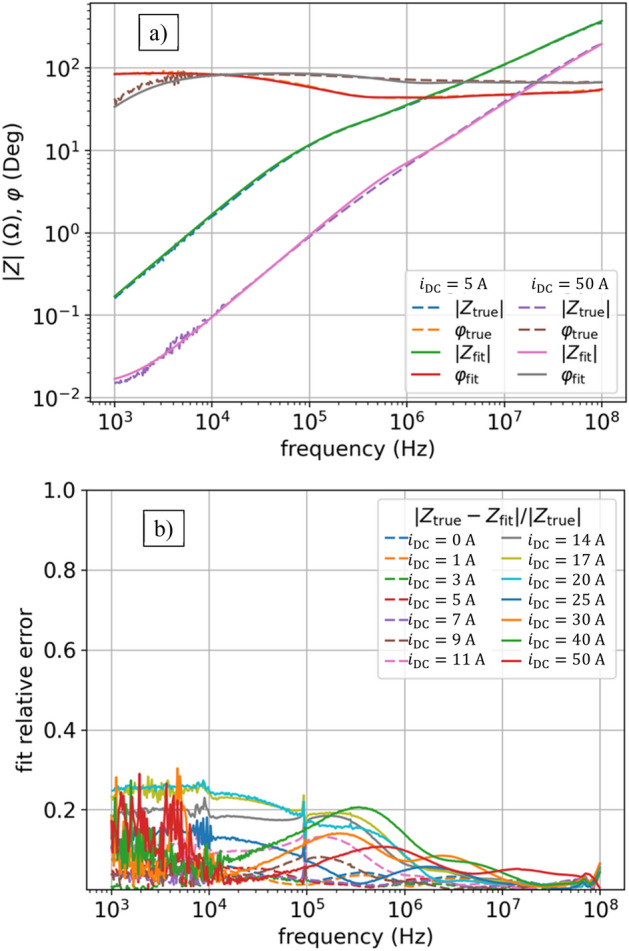
DE results of impedance $Z(f,{i}_{\mathrm{DC}})$ fitting for model (6): (**a**) fit vs true values of $\left|Z\right|\left(f,{i}_{\mathrm{DC}}\right)$ and $\varphi (f,{i}_{\mathrm{DC}})$ for two currents: ${i}_{\mathrm{DC}}=5$ A, ${i}_{\mathrm{DC}}=50$ A; (**b**) relative error as per (4) for all currents. Now using DE metaheuristic optimization method that gives best fit with lowest error, much better than standard NLS (compare with Fig. 5).

**Figure 9:**
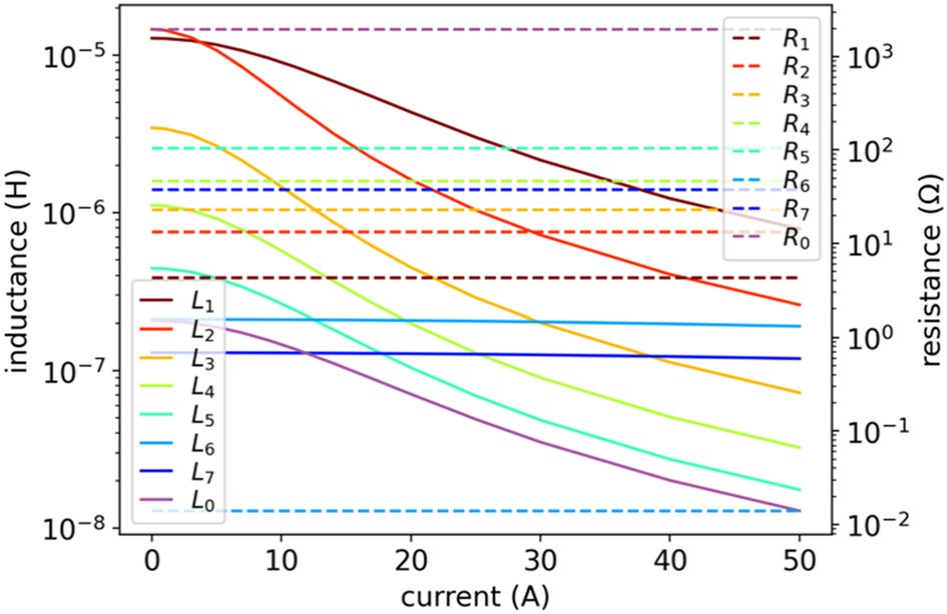
DE results of inductances $L(i)$ given by (5) and constant resistances $R(i)$ in model (6).

**Figure 10:**
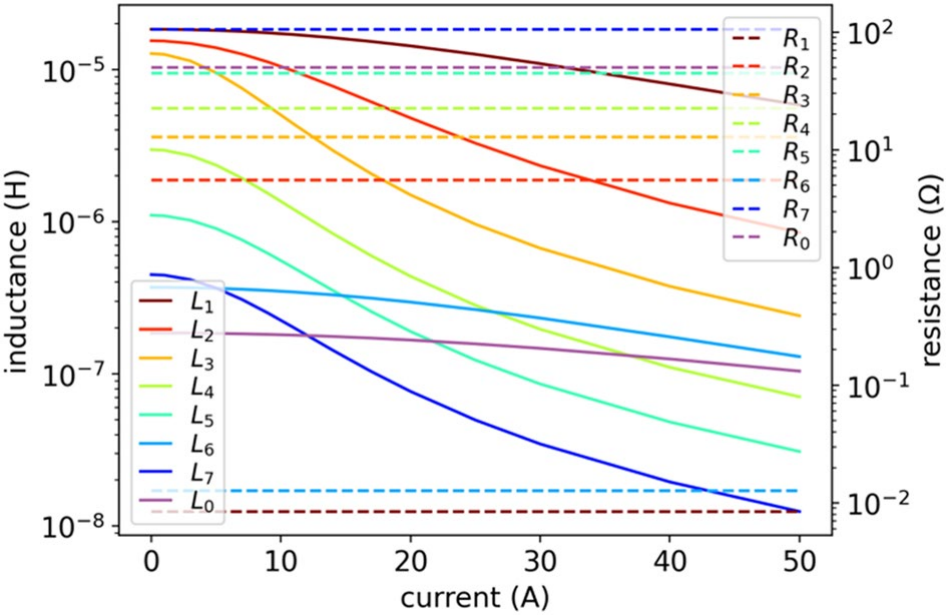
DE results of inductances $L(i)$ given by (5) and constant resistances $R(i)$ in model (6). Another Results for fitting model (6) with DE optimization, similar courses as in Fig. 9 but now a bit more ordered.

DE optimizes a problem by maintaining a population of candidate solutions and creating new candidate solutions by combining existing ones, and then keeping that candidate solution which offers the best fit to the optimization problem. Figure 8 shows the results of the DE fit, which are superior (see also Table 1). The DE is the best method we have tested. In terms of fit accuracy, it is even better than the original model with a much greater number of parameters as proposed in^10^. Additionally, it provides smooth (physical) characteristics of $L(i)$ and $R(i)$ parameters – see Figs. 9 and 10, which is not provided by the model introduced in^10^. The following hyperparameters of the DE algorithm were used: *population size* $=300$, mutation constant = 0.7, *crossover probability* $=0.8$, and *best1bin* strategy^32^. Figure 10 shows fit results for another run of the DE algorithm with the same parameters – now the characteristics are slightly different. This shows that different runs of the algorithm results in slightly different parameter sets for the model and therefore slightly different matches. This is a characteristic feature of probabilistic optimization methods.

### Annealing

**Figure 11:**
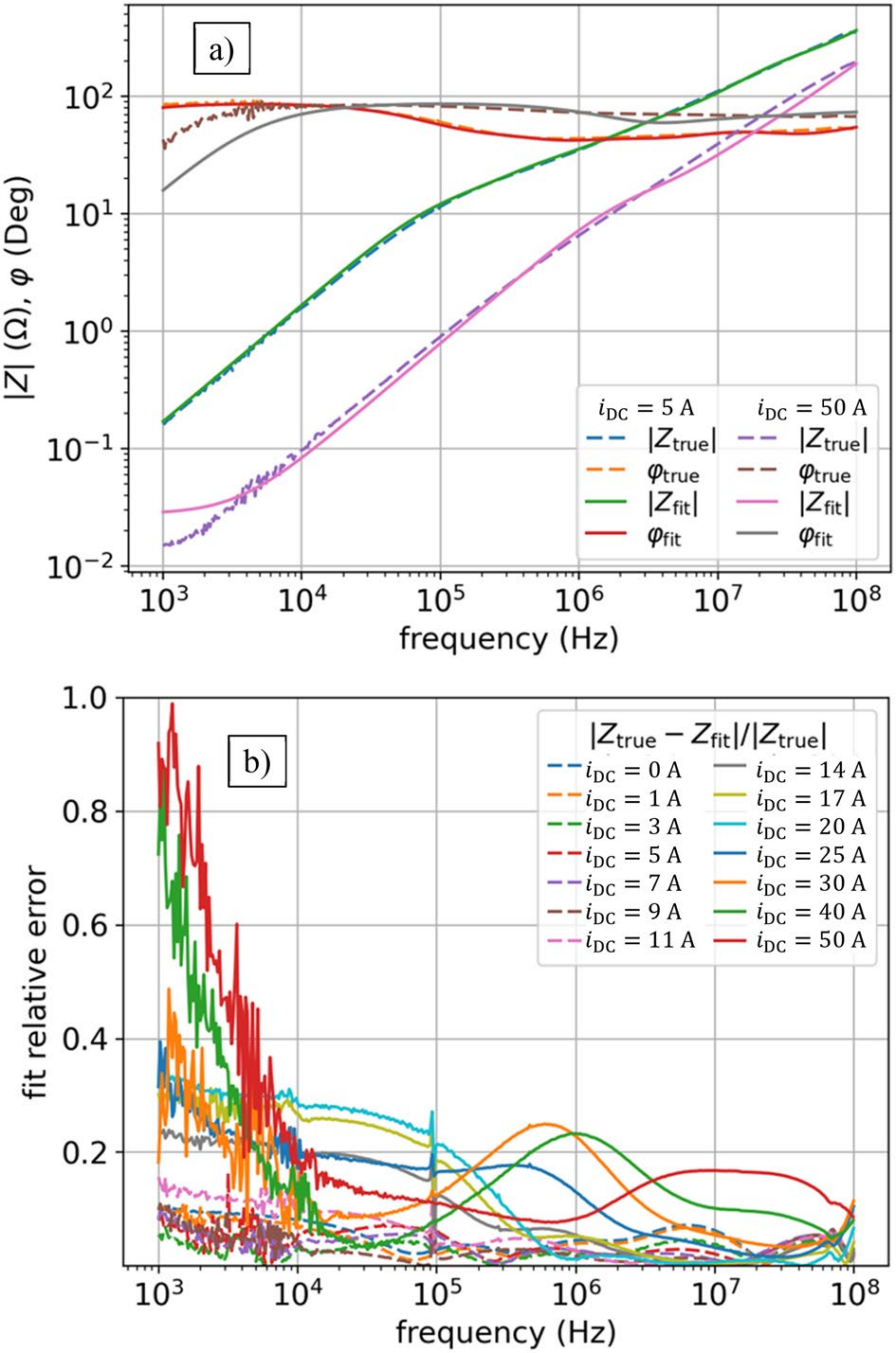
DA results of impedance $Z(f,{i}_{\mathrm{DC}})$ fitting for model (6): (**a**) fit vs true values of $\left|Z\right|\left(f,{i}_{\mathrm{DC}}\right)$ and $\varphi (f,{i}_{\mathrm{DC}})$ for two currents: ${i}_{\mathrm{DC}}=5$ A, ${i}_{\mathrm{DC}}=50$ A; (**b**) relative error as per (4) for all currents. DA gives a bit worse results on model (6) than DE (compare with Fig. 8).

Now we will compare the results obtained for soft-computing evolutionary metaheuristics with more traditional approach: simulated annealing, for which we chose modern implementation of Dual Annealing (DA) ^31^. The DA combines the generalization of Classical Simulated Annealing (CSA) and Fast Simulated Annealing (FSA) coupled to a strategy^33^ applied for local searching on accepted locations. The results are slightly worse than for DE (see Table 1), especially for lower frequencies – see Fig. 11. The DA had similar problem to PSO with finding a fit in a wide range of currents. The parameters applied: *initial temperature* $=20000$, *visit parameter* $=2.8$, and *acceptance parameter* $=-100$.

**Figure 12:**
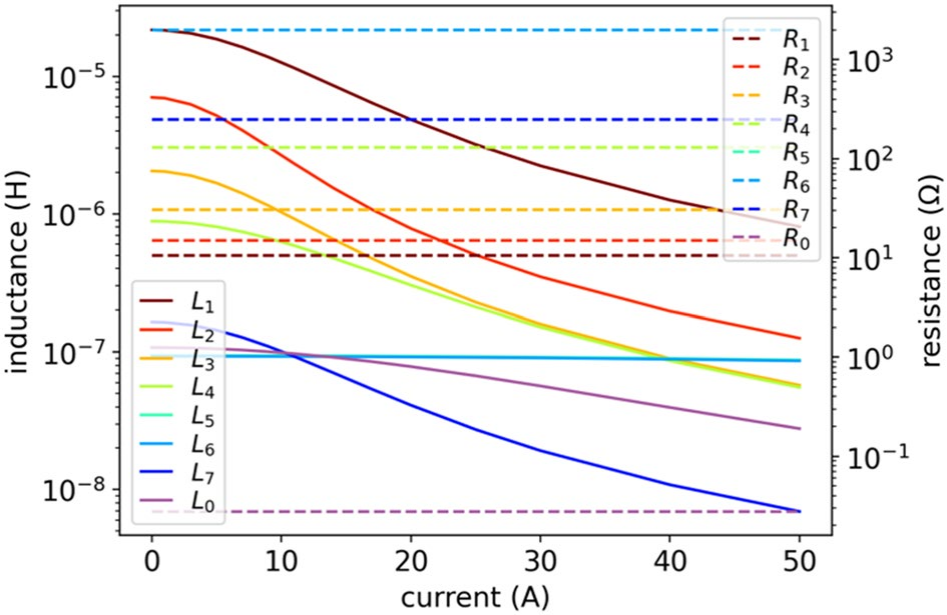
DA results of inductances $L(i)$ given by (5) and constant resistances $R(i)$ in model (6).

## JA Models

**Figure 13:**
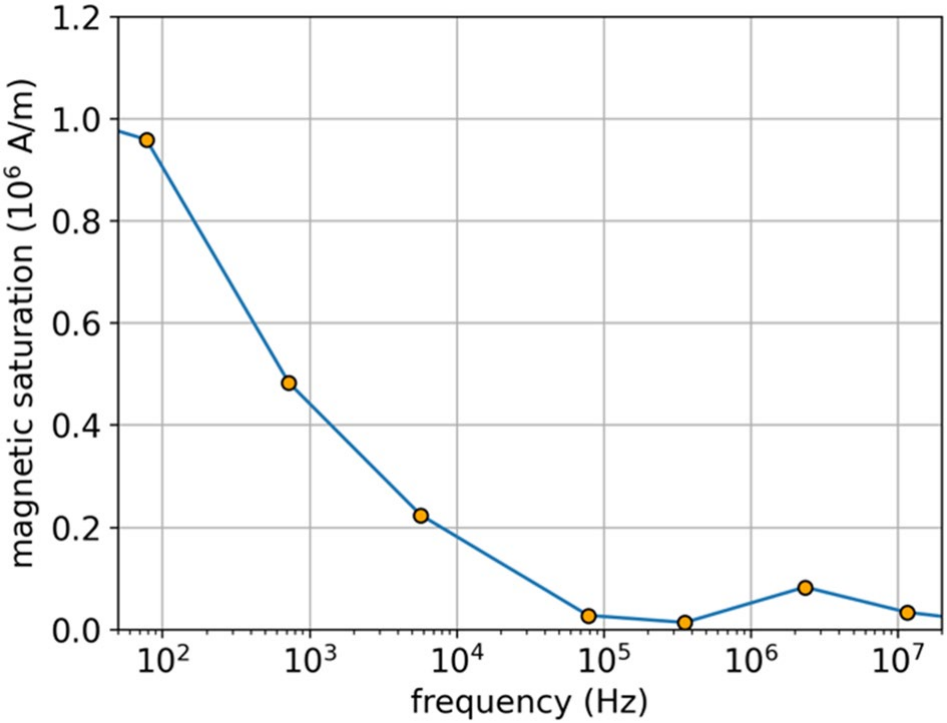
Magnetic saturation ${M}_{s}$ across frequencies that may enter JA model to make it frequency dependent.

Finally, there is one more advantage of taking the fit of the current characteristics for inductance $L(i)$ in the form of the Langevin derivative (5): thanks to this, we obtain the frequency characteristics of magnetic saturation ${M}_{s}$, directly proportional to ${l}_{0}$ in (5), as presented in Fig. 13.

Frequency-dependent ${M}_{s}$ enters JA and makes it frequency dependent. We also checked the variability of $a$ parameter from (5) and it remains almost fixed ($\pm 10\%$) which is physically justified. The attempts of the frequency dependent JA model have been reported^34,35^, however, our method makes it possible to derive the frequency dependence of the parameter that enters JA and hence we can naturally/easily get the frequency dependent JA.

## Summary

In this work, we presented a method of creating lumped element equivalent circuit (LEEC) model of magnetic rings that can be implemented in EMTP or SPICE alike simulators for performing power system transient studies. We presented the method of selecting model parameters with the use of AI metaheuristic optimization algorithms.

Three AI metaheuristic optimization algorithms were tested: Particle Swarm Optimization (PSO), Differential Evolution (DE) evolutionary algorithms, and modern implementation of simulated Dual Annealing (DA). The DE algorithms proved to be effective in terms of fitting accuracy of the model parameters for the whole range of frequency and currents. The models were tested based on the measured impedance characteristics for frequency up to 100 MHz and current up to saturation.

We showed that the proposed method works on the actual measurements, i.e., it gives good fit and smooth current characteristics of the parameters. We presented that the parameters (inductances $L$ and resistances $R$) behave physically, i.e., $L$ decreases with the current, and $R$ is relatively constant, which shows that our model is not only heuristic, but has a physical justification. As a byproduct, we were able to determine the frequency dependence of the magnetic saturation ${M}_{\mathrm{S}}$ parameter, which can be directly inserted to Jiles-Atherton (JA) model, and therefore it is easy to obtain the JA extension to the frequency domain, which can be useful in many applications. The Langevin function used in this work has a physical interpretation, which reduces number of parameters in the model.

The presented method is universal, and the selection of the rings used in the study was dictated by their reference to the actual and significant areas of application. Introducing further (apart from Langevin) physical dependencies to the model (e.g. taking into account geometry, size, or material) could be beneficial, as it could reduce the number of model parameters. The open question, in this case, is whether we would still be able to optimize such a model (find its parameters with sufficient accuracy) using the methods described in the paper.

## Data Availability

The codes used to calculate the results of this study are available from the corresponding author upon reasonable request.
